# Selective Extraction of Diazepam and Its Metabolites from Urine Samples by a Molecularly Imprinted Solid-Phase Extraction (MISPE) Method

**DOI:** 10.3390/polym16050635

**Published:** 2024-02-27

**Authors:** Ana María Gil Tejedor, Juan Carlos Bravo Yagüe, Gema Paniagua González, Rosa María Garcinuño Martínez, Pilar Fernández Hernando

**Affiliations:** Department of Analytical Sciences, Faculty of Sciences, National Distance Education University (UNED), Urbanización Monte Rozas, Avda. Esparta s/n, 28232 Las Rozas, Madrid, Spain; ana.gil@correo.gob.es (A.M.G.T.); rmgarcinuno@ccia.uned.es (R.M.G.M.); pfhernando@ccia.uned.es (P.F.H.)

**Keywords:** benzodiazepines, molecular imprinting solid-phase extraction, urine samples, HPLC-DAD

## Abstract

In this research, a molecularly imprinted polymer (MIP) was synthesized by precipitation polymerization using oxazepam (OZ) as a template molecule and was subsequently applied as a selective sorbent for the extraction of diazepam (DZP) and its metabolites in urine samples using an SPE cartridge. OZ, temazepam (TZ), nordiazepam (NZ) and DZP were analyzed in the final extracts by high-performance liquid chromatography with diode array detection (HPLC-DAD). The SPE extraction steps were optimized, and the evaluation of an imprinting factor was carried out. The selectivity of the method for OZ versus structurally related benzodiazepines (BZDs), such as bromazepam (BRZ), tetrazepam (TTZ) and halazepam (HZ), was investigated. Under the optimum conditions, the proposed methodology provided good linearity in the range of 10–1500 ng/mL, with limit of detection values between 13.5 and 21.1 ng/mL and recovery levels for DZP and its metabolites from 89.0 to 93.9% (RSD ≤ 8%) at a concentration level of 1000 ng/mL. The proposed method exhibited good selectivity, precision and accuracy and was applied to the analysis of urine samples from a real case of DZP intake.

## 1. Introduction

Over the past few years, the misuse and abuse of benzodiazepines (BZDs) has become a health issue [1,2]. BZDs are amongst the most widely used medicines, mainly to treat anxiety and insomnia, and have other uses as anticonvulsants and anesthetics. However, they can also produce several problems when they are used improperly—for example, when they are consumed for a longer time frame than prescribed, with the risk of developing a physical dependence on these drugs [3], or when they are consumed in combination with other substances, such as alcohol or opiates [4,5,6]. As prescription medicines, they can affect the ability of consumers to drive safely [7,8] or increase the risk of falls, leading to fractures in older persons [9]. Illegal uses include criminal purposes like rape and robbery, in which these drugs can be masked in drinks or food [10,11]. Moreover, the concomitant use of BZDs and opioids has been associated with an increased risk of overdose and overdose mortality [12,13,14].

Despite their risks, the use of BZDs has not fallen; it even increased during the COVID-19 pandemic due to anxiety, insomnia, and depressive disorders [15,16,17,18]. Therefore, to prevent BZDs abuse, the development of accurate and highly sensitive methods for the determination of these is of great importance.

Diverse analytical techniques have been used for the analysis of BZD drugs in biological matrices: capillary electrophoresis–tandem mass spectrometry (CE-MS/MS) [19], GC-MS/MS [20,21], HPLC-MS/MS [22,23,24], HPLC-DAD [25,26], differential pulse voltammetry [27] and voltametric electronic tongues [28].

Previously, sample treatment was necessary for the determination of analytes; thus, diverse sample preparation methods, mainly liquid–liquid extraction (LLE) [29,30], solid-phase extraction (SPE) [31,32,33] and solid-phase microextraction (SPME) [34,35,36], have been established for the extraction of BZDs from biological matrices.

DZP is one of the most prescribed BZDs and is commonly used to treat diverse conditions, such as convulsions, insomnia and anxiety. In 2020, DZP was among the ten most used medications in Europe [37]. In the human body, DZP is metabolized by the liver into NZ, TZ and OZ, which are then eliminated by the kidney. For this reason, as well as advantages such as simplicity and non-invasiveness of collection, urine analysis is the preferred sampling method for DZP determination [38,39].

For urine analysis, SPE has been broadly used for sample pretreatment, due to its simplicity, low solvent consumption and high extraction efficiency [40,41], allowing for the separation of target analytes from interferents prior to determination. Nevertheless, when SPE is used, the target analyte is extracted as well as other compounds that can be retained by the sorbent. However, the use of selective sorbents, such as those developed from molecularly imprinted polymers (MIPs), could provide the ability to recognize and extract only the target molecules among different compounds. The molecular imprinting technology allows designed sorbents to specifically adsorb an analyte or group of analytes of interest [42]., A porous, highly cross-linked polymeric net that can specifically recognize the template analyte in the molecular imprinting is obtained. First, functional polymerizable monomers form a pre-polymerization complex with the template molecule. After the addition of a large percentage of a cross-linker monomer, as well as an initiator compound, a porous, polymeric matrix is obtained. When the template is eliminated, cavities complementary to the template molecule are formed and have recognition properties selective to the original template molecule [43,44].

In recent times, some analytical methodologies have been developed using MIPs to selectively extract DZP and/or its metabolites in biological samples such as hair [45], blood serum [46,47] and urine [48,49]. However, none of them have been able to efficiently carry out the simultaneous extraction of DZP and its three metabolites (NZ, TZ and OZ).

The aim of this work was to develop a MISPE-HPLC-DAD method for the extraction and determination of DZP and its main metabolites in urine. Once the MISPE parameters were established, the method was used for the extraction of DZP, OZ, TZ and NZ in spiked and real urine samples.

## 2. Experimental Procedure

### 2.1. Reagents and Materials

DZP, OZ, TZ and NZ were provided by EDQM (Strasbourg, France). TTZ, HZ and BRZ were obtained from pharmaceutical tablets. Flunitrazepam as internal standard (IS) was acquired from Sigma-Aldrich (St. Louis, MO, USA). A chemical grade of the highest purity was used for all solvents and chemicals, which were obtained from different manufacturers: methanol, acetonitrile, acetic acid, hydrochloric acid and sodium acetate from Scharlau (Barcelona, Spain); 2,2-azo(bis)-isobutyronitrile (AIBN) and β-glucuronidase (type HP-2 from *Helix pomatia*) from Sigma-Aldrich (St. Louis, MO, USA); and ethylene glycol dimethacrylate (EGDMA) and methacrylic acid (MAA) from Merck (Darmstadt, Germany).

Stock solutions of the individual BZDs and flunitrazepam in acetonitrile were prepared at a 1000 mg/L concentration. A working solution containing a mixture of the four BZDs (DZP, OZ, NZ and TZ) was prepared in acetonitrile at a 100 mg/L concentration. Stock solutions were transferred into amber-colored flasks and stored at −18 °C.

### 2.2. Synthesis of Molecularly Imprinted Polymer

A MIP was synthesized by precipitation polymerization, using OZ as a template molecule, with a molar ratio of template (OZ): functional monomer (MAA): crosslinker (EGDMA) of 1 (0.08 mmol): 4 (0.32 mmol): 20 (1.6 mmol). After mixing the reagents with acetonitrile in a test tube, a certain amount of AIBN as an initiator was added; then, the solution was sonicated for 5 min and purged with oxygen-free nitrogen for 10 min. The test tube was sealed and placed in a water bath at 55°C for 24 h to carry out the polymerization reaction. A non-imprinted polymer (NIP), as a control polymer, was prepared in the same way, without a template (OZ). After the polymerization process, the obtained imprinted and non-imprinted particles were collected on a nylon membrane filter by using a vacuum filtration system. The remaining template was removed using an acetic acid–methanol solution via the Soxhlet extraction method. Then, the polymeric particles were completely dried in an oven at 60 °C and subsequently stored in a desiccator prior to use as sorbents in the MISPE procedure.

### 2.3. Urine Sample Treatment

An early morning urine sample was collected from a volunteer who was prescribed DZP (Valium^®^) at a nightly dosage of 5 mg. This volunteer was a smoker and was being treated for several diseases, including chronic obstructive pulmonary disease. The samples were collected in urine container cups and then transferred to 2.5 mL plastic tubes with no preservative and finally stored at −20 °C.

The enzymatic hydrolysis of the urine samples was carried out using a modified protocol [50]. A volume of 50 µL of acetate buffer (pH 4.5, 2 M) and 50 µL of β-glucuronidase enzyme were added to 1 mL of the urine sample in a 10 mL Teflon tube. The samples were placed in a water bath at 56 °C for 1.5 h to carry out the hydrolysis reaction. After this time, any precipitate formed from the hydrolyzed sample was removed by centrifugation at 10,000 rpm for 10 min, preventing the blocking of the MISPE cartridge. Consequently, a solution of acetonitrile and acetate buffer (pH 4.5, 0.1 M) was added to an aliquot of 1 mL of urine. The solution was mixed for 1 min in a vortex mixer and an aliquot of 0.5 mL of this mixture was subjected to the MISPE procedure.

### 2.4. MISPE Procedure

An amount of 50 mg of MIP-synthesized microspheres were packed in empty polypropylene SPE cartridges with the aid of a vacuum manifold. An identical process was used to fill the NIP’s SPE cartridge. A solution of acetic acid–methanol (20:80, *v*/*v*) followed by acetonitrile was applied to wash the cartridge located in the vacuum system manifold (Vac Elut 20, provided by Agilent, Madrid, Spain). The conditioning step consisted of 2 × 0.5 mL acetonitrile followed by 0.5 mL of a mixture 30:70 (*v*/*v*) of acetonitrile and acetate buffer (pH 4.5, 0.1 M). After loading a volume of 0.5 mL preconditioned urine, a washing step with 0.5 mL of the mixture (25:75, *v*/*v*) of acetonitrile and acetate buffer (pH 4.5, 0.1 M), followed by 0.5 mL of water, was applied to the cartridges. Lastly, an elution step was carried out by adding 2 × 0.5 mL of acetic acid–methanol (20:80, *v*/*v*) solution to extract the retained analytes. The eluates were collected and spiked with 20 μL of a solution of IS at a 100 mg/L concentration and evaporated to dryness under a stream of nitrogen in a solvent evaporation sample concentrator at 45 °C. The dry residue was reconstituted with 250 µL of the mobile phase and a volume of 20 µL was injected into the chromatographic system to separate and detect the selected compounds.

### 2.5. Instrumental Analysis

The chromatographic analyses were carried out using an Agilent 1200 series LC system (Agilent Technologies, Madrid, Spain) equipped with a diode array detector and an autosampler with a 20 µL loop. The instrumental parameters were controlled via the Agilent ChemStation software Rev. B. 04. 02. 96. A ZORBAX Eclipse Plus C18 column (10.0 cm × 4.6 mm i.d.; 3.5 µm particle size) was used for the separation of the compounds, using an isocratic elution method at 25 °C, with a composition of 47% methanol, 7% acetonitrile and 46% water at a flow rate of 1.0 mL/min. The wavelength of 230 nm was used for HPLC-DAD detection.

## 3. Results and Discussion

### 3.1. Optimization of MISPE Procedure

A MISPE procedure based on an aqueous medium was developed to extract DZP and its main metabolites, OZ, NZ and TZ, from urine samples with minimal pretreatment. The steps of the MISPE procedure (loading, washing and elution) were optimized with the aim of enhancing the MIP’s recognition of the analyte, OZ.

#### 3.1.1. Loading Step

**Figure 1:**
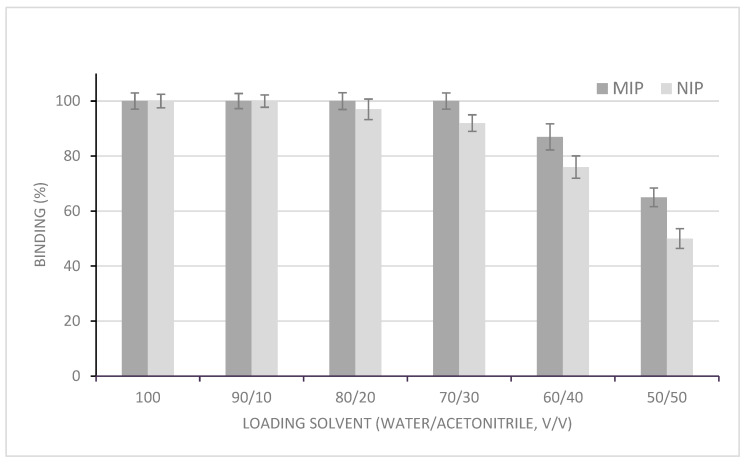
Effect of water and acetonitrile content in the binding capacity of OZ by MIP and NIP cartridges in loading step (*n* = 5).

Considering the difference between the polymerization medium (acetonitrile) and the sample, studies of the binding of OZ in the MIP and NIP were carried out using different solutions of OZ in water–acetonitrile at several solvent ratios, as shown in Figure 1. The sample mixture was percolated through the SPE cartridge after conditioning with 2 × 0.5 mL of acetonitrile and 0.5 mL of acetonitrile–water solution, with a similar ratio as used in the loading step. The assays were performed in quintuplicate. With these conditions, OZ was fully retained on both polymers (MIP and NIP) in aqueous solutions up to a 10% of acetonitrile, due to the predominance of non-specific hydrophobic interactions. An increase in acetonitrile to 30% led to the total retention of OZ by the MIP, whereas the bleeding of OZ was observed for the NIP. The increase in the acetonitrile percentage reduced the amount of OZ retained in both polymers, being more prominent for the NIP; thus, a mixture of acetonitrile–water (30:70, *v*/*v*) was chosen as the loading solvent.

The effect of the pH on the loading step was also studied within the range of 4.0 to 6.5, using 0.5 pH increments, employing an acetonitrile–acetate buffer solution (0.1 M). The retention of OZ by the MIP slightly decreased at pH 5.0. Consequently, a solution consisting of acetonitrile–acetate buffer (pH 4.5, 0.1 M) in a ratio of 30:70 (*v/v*) was selected as the loading solvent.

Loading volumes ranging from 0.5 mL to 2 mL in 0.5 mL increments were studied to determine the maximum sample breakthrough volume. The results revealed leakage from the MIP when volumes higher than 0.5 mL were used. Therefore, a loading volume of 0.5 mL was selected as the optimum one.

#### 3.1.2. Washing Step

The washing solvent plays a crucial role in the MISPE procedure, aiming to maximize the specific interactions between the analyte and binding sites while eliminating non-specific interactions by removing matrix components from the cartridge. To optimize the washing solution, several solvents were tested, including an acetate buffer (pH 4.5, 0.1 M), water and an acetate buffer with increasing volumes of acetonitrile, using a loading volume of 0.5 mL. 

In this study, the MIP behaved like a reverse-phase sorbent when loading the aqueous samples, and the target analytes were retained by non-specific interactions. Therefore, it was necessary to apply a washing process capable of removing analytes that were non-specifically bound to the selective imprinted polymer [51,52]. Water used as a washing solvent resulted in the slight bleeding of OZ, so mixtures of acetonitrile–acetate buffer (pH 4.5, 0.1 M) were considered as washing solvents. An increase in the percentage of acetonitrile led to the negligible washing of OZ from the imprinted polymer, up to a mixture ratio of 75:25 (*v*/*v*). In comparison to the NIP, the bleeding of OZ was consistently higher regardless of the solvent used. This bleeding was particularly remarkable with mixtures of acetonitrile–acetate buffer with proportions higher than 20:80 (*v*/*v*). Thus, a mixture of 25:75 (*v*/*v*) of acetonitrile–acetate buffer (pH 4.5, 0.1 M) was selected as the optimum solvent to carry out selective washing, given the markedly different behavior of both polymers. A final washing step using water was used to remove any salt from the matrix.

#### 3.1.3. Elution Step

The choice of a suitable elution solvent is important as it is responsible for the efficient desorption of the target analyte from the cartridge. A small amount of a modifier, such as a weak acid, is frequently added to aid in breaking the hydrogen bonds to recover strongly bound analytes. The elution solution was optimized by using fractions of 0.5 mL of methanol and mixtures of methanol–acetic acid. The elution of OZ with methanol was not complete, even when using two volumes of 0.5 mL. However, the elution efficiency increased with the addition of acetic acid. Total cumulative elution was achieved when a 2 × 0.5 mL elution step with an acetic acid–methanol (20:80, *v*/*v*) solution was applied. The polarity of this elution solvent was sufficient to disrupt the ionic and hydrogen bonds established between the analyte and the MIP. Increasing the ratio of acetic acid produced no advantage since it was necessary to use two volumes of 0.5 mL to reach 100% elution.

### 3.2. Imprinting Factor and Specific Adsorption

**Figure 2:**
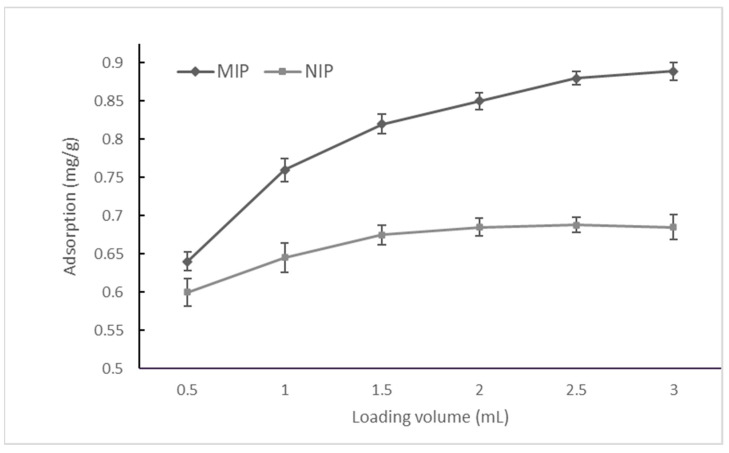
Adsorption of OZ (100 mg/L in acetonitrile) onto imprinted and non-imprinted polymers.

Sequential loads of the OZ solution at 100 mg/L (in acetonitrile), in 0.5 mL increments, were performed in the imprinted and non-imprinted SPE cartridges to saturate the binding sites. The percolated solution was collected and analyzed to calculate the amount of OZ adsorbed by the imprinted and non-imprinted polymers. After loading a total volume of 2.5 mL of OZ solution, the MIP and NIP cartridges showed an OZ adsorption capacity of 0.89 and 0.65 milligrams of analyte per gram of polymer, respectively. The specific adsorption assays were carried out in quintuplicate (see Figure 2).

The imprinting factor (IF) is a measure of the strength of the interaction of the imprinted polymer with the template molecule and it was studied in the washing step. The IF was calculated as the ratio between the amount of analyte bound to the imprinted and non-imprinted polymers (Equation (1)), denoted by Q_MIP_ and Q_NIP_ [53], respectively. Q describes the difference between the initial and final amounts of the analyte in the solution mixture (Equation (2)), where C_i_ and C_f_ are the initial and final concentrations of the analyte in the solution, m is the mass of the polymer, and V is the volume of the analyte solution mixture, respectively.

IF = Q_MIP_/Q_NIP_ (1)

Q (mol/g) = (C_i_ − C_f_)·V/m (2)

The cartridges of the MIP and NIP were washed with sequential volumes of 0.5 mL of 25:75 (*v*/*v*) acetonitrile–acetate buffer (pH 4.5, 0.1 M) solution. The results showed the gradual elimination of the non-specific adsorption of OZ on both polymers, giving an amount of OZ residually bound to the NIP and MIP of 0.069 and 0.138 mg/g of polymer, respectively, with an IF of 2.0. This demonstrates that the MIP retained a larger amount of the analyte compared to the NIP.

Specific adsorption of 0.069 mg/g of OZ on the MISPE cartridge was calculated as the difference between the OZ concentrations residually bound on the MIP and NIP cartridges after extensive washing.

### 3.3. Analytical Performance

The linearity was established by using calibration curves for DZP, NZ, OZ and TZ, prepared from blank urine samples spiked with known amounts of stock solutions. Urine samples were treated according to the previously described protocol to achieve a working range of 10–1500 ng/mL and then subjected to the MISPE procedure (Section 2.4). The ratio of the analyte signal to the internal standard signal was plotted against the concentration of the studied BZDs. Linear least-squares regression was employed to fit the data to a linear calibration curve.

The blank urine samples were spiked at 10, 15, 20 and 30 ng/mL concentrations of OZ, TZ, NZ and DZP and subjected repeatedly (*n* = 5) to the MISPE procedure. The detection and quantification limits were calculated from the standard deviation of the lowest measurable concentration, for a signal-to-noise ratio of 3 and 10, respectively. The results are summarized in Table 1.

To assess the accuracy in terms of the recovery efficiency of both polymers, drug-free urine samples spiked with each BZD at two different concentration levels (250 and 1000 ng/mL) were subjected to the sample treatment protocol as previously detailed, followed by the MISPE procedure. Recovery rates were calculated by spiking the extracts of MISPE-subjected drug-free urine samples at 250 and 1000 ng/mL concentration levels of BZDs and comparing the ratios of the peak areas of each analyte vs. the internal standard. The recovery obtained by the MIP ranged from 89.1 to 99.4%, which was higher than that obtained by the NIP, which ranged from 66.5 to 77.7% (Table 2). The difference observed in the extraction efficiency could be ascribed to the selective washing step.

### 3.4. Selectivity of MISPE Procedure

The selectivity of the proposed method for OZ versus structurally related BZDs such as BRZ, TTZ and HZ was investigated by subjecting spiked urine samples (1000 ng/mL of these compounds to the MISPE procedure. The recovery percentages and the relative standard deviations for each analyte are given in Table 3.

**Figure 3:**
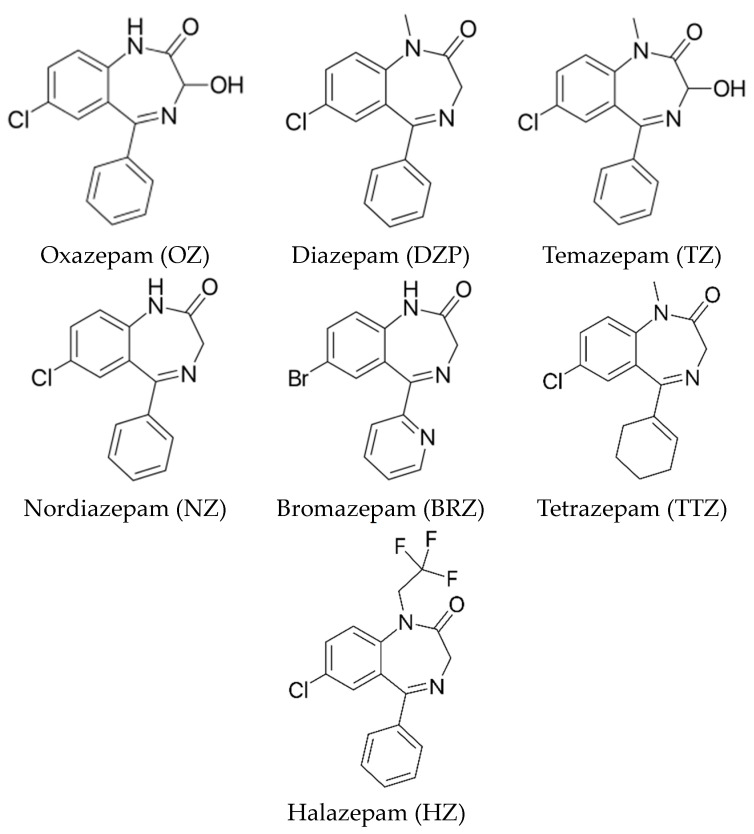
Molecular structures of the BZDs studied.

DZP showed the highest recovery by the MISPE cartridge (94.0%), followed by OZ (91.5%), TZ (90.8%) and NZ (89.1%). Considering that OZ was the template molecule used in the molecular imprinting procedure, it was expected to obtain the highest recovery. Nevertheless, specific interactions between the BZDs and recognition sites, created during imprinting polymerization, were not promoted in the loading step by using a similar solvent as in the polymerization but in the washing step using a 25:75 (*v*/*v*) acetonitrile–acetate buffer (pH 4.5, 0.1 M) solution. In this environment, a certain degree of hydrophobic interaction persisted, which could explain why DZP was the BZD with the highest recovery throughout the analysis. On the other hand, the results indicated that BRZ, a widely modified BZD (7-bromine substituent and 5-pyridyl instead of 5-phenyl ring), obtained the lowest recovery (50.0%) following the MISPE protocol. Furthermore, when the benzene is replaced by a cyclohexadiene, in the case of TZ, or the amino group (-NH) of cycloheptane is replaced by a trifluoroethyl group (HZ), the binding is also reduced (Table 3). This effect can be attributed to the absence of hydrogen bonding or to steric effects, possibly caused by bulkier chains in the case of HZ. The molecular structures of the studied BZDs are shown in Figure 3.

In the current literature, DZP MIPs against DZP were synthesized and applied to extraction from human plasma [46,47] Other studies with MIPs have been carried out with OZ and NZ as templates, respectively [48,49], to selectively extract OZ in the first case and OZ, BRZ and alprazolam in the second case. Our method showed greater selectivity towards DZP and its metabolites, enabling the simultaneous selective extraction of these four compounds. The main advantage of the proposed method is the capability to perform the selective and simultaneous extraction of DZP and its main metabolites. Table 4 shows a comparison between the results of this work and those obtained with other methods for the determination of BZDs in biological fluids.

### 3.5. Analysis of Urine Sample

**Figure 4:**
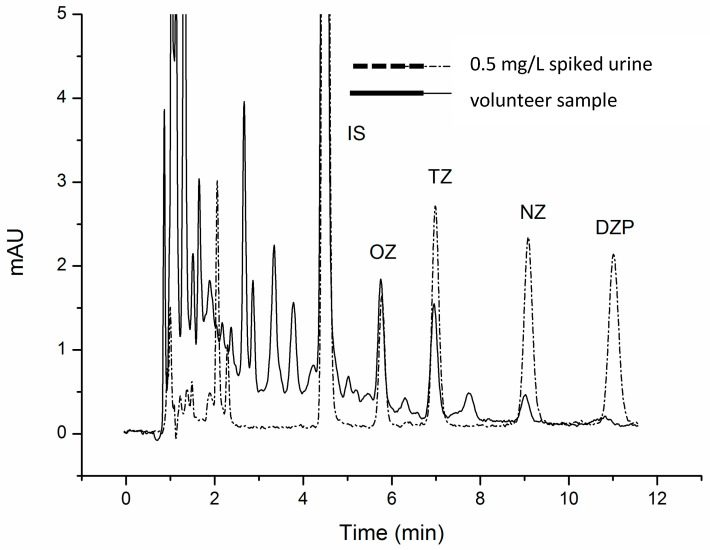
Chromatograms of spiked urine sample with 500 ng/mL of OZ, TZ, NZ and DZP and urine from volunteer, both subjected to MISPE procedure and enzymatic hydrolysis.

The optimized MISPE procedure was applied to analyze a sample collected from a volunteer with a long-term nightly prescription of 5 mg of DZP (Valium^®^), following the protocol described in Section 2.4. The concentrations obtained in the urine for OZ, TZ, NZ and DZP were 62.2 ± 7.5, 35.5 ± 4.0, 82.0 ± 1.0 and 56.0 ± 8.0 ng/mL, respectively. Figure 4 shows the comparison between the urine from the DZP-prescribed volunteer and a drug-free urine sample spiked with 0.5 mg/L of these BZDs, both subjected to the proposed method.

DZP is metabolized via N-demethylation by hepatic enzymes, leading to the formation of its major active metabolite, NZ (desmethyldiazepam), and hydroxylated to the minor active metabolite TZ. NZ is further metabolized to OZ via hydroxylation, and TZ can be in turn demethylated to OZ or glucuronidated. OZ and TZ are primarily eliminated by glucuronidation. Consequently, DZP is excreted in the form of glucuronides of its main metabolite, OZ, and, to a lesser extent, TZ, with the minimal excretion of the unmetabolized drug in the urine [54,55,58,59]. Therefore, the obtained results were consistent with the literature.

## 4. Conclusions

A new MIP using OZ as the template molecule has been synthesized and applied for the selective solid-phase extraction of OZ, TZ, NZ and DZP from urine samples. High-performance liquid chromatography with diode array detection (HPLC-DAD) was chosen as the analytical technique for the analysis of DZP and its metabolites due to their simplicity, cost-effectiveness, and reliability, making them a good choice for quantitative analysis and BZD separation. In addition, the HPLC-DAD technique employed for the development of the analytical method enabled, as the results demonstrated, the reliable separation, detection, and quantification of the studied analytes in the selected matrix, with appropriate sensitivity, selectivity, precision, and accuracy. The recovery rates at different spiking levels in the proposed MISPE method for the studied analytes were established to be higher than 89.1%, and high sensitivity was achieved, with detection limits between 13.5 and 21.1 ng/mL. The retention behavior of DZP and its metabolites evidenced the molecular recognition in the MISPE procedure.Poor recovery (50.4–63%) was obtained for BRZ, TTZ and HZ, whose molecular structures did not fit well within the imprinting cavities, indicating that the imprinted polymer exhibited selective retention only for DZP and its metabolites. The developed method was successfully applied to the extraction OZ, TZ, NZ and DZP from a real urine sample.

## Figures and Tables

**Table 1:** Analytical characteristics of the optimized MISPE-HPLC-DAD methodology for BZD analyses in human urine samples.

Analyte	Linear Equation	R	LOD (ng/mL)	LOQ (ng/mL)
OZ	y = 0.219x + 0.0012	0.9984	16.2	53.5
TZ	y = 0.4179x − 0.0026	0.9982	21.1	63.9
NZ	y = 0.4576x + 0.002	0.9993	13.5	44.5
DZP	y = 0.4823x − 4 × 10^−5^	0.9985	21.0	69.3

**Table 2:** Comparison of accuracy (% recovery; *n* = 5) and repeatability (RSD; *n* = 5) obtained for DZP, NZ, TZ and OZ on the MIP and the NIP cartridges in human urine samples at spiking levels of 250 and 1000 ng/mL.

Analyte	1000 ng/mL				250 ng/mL			
	MIP		NIP		MIP		NIP	
	Recovery	RSD	Recovery	RSD	Recovery	RSD	Recovery	RSD
OZ	91.5	5.7	67.3	14.2	92.4	2.4	66.5	8.8
TZ	90.8	6.0	72.1	10.4	91.4	5.3	74.8	3.7
NZ	89.1	7.9	72.9	10.1	89.5	8.1	70.1	6.6
DZP	94.0	4.7	77.7	8.9	99.4	4.3	76.7	3.7

**Table 3:** Accuracy (% recovery; *n* = 5) and repeatability (RSD; *n* = 5) obtained for seven related BZDs via the MISPE procedure in human urine samples (*n* = 5) at a concentration of 1000 ng/mL.

Analyte	Recovery (%)	RSD (%)
OZ	91.5	5.7
TZ	90.8	6.0
NZ	89.1	7.9
DZP	94.0	4.7
BRZ	50.0	2.1
HZ	69.1	2.0
TTZ	63.4	1.8

**Table 4:** Comparison of the proposed method with other methods for BZDs determination.

Sample Matrix	Method of Analysis	Template	Recovery (%)	Linear Range (ng/mL)	Det. Limit (ng/mL)	Quant. Limit (ng/mL)	Ref
Blood serum	MISPE + HPLC-UV	DZP	DZP 95.3	-		DZP 3.5	[46]
Blood serum	MISPE + HPLC-UV	DZP	DZP 105.6	-	-	-	[47]
Urine	MIP + HPLC-DAD	OZ	OZ 88	2–600	OZ 0.5	OZ 2.0	[48]
Urine	MISPE + HPLC-MS	NZ	-	-	-	OZ 0.357	[49]
Urine	MISPE + HPLC-DAD	DZP	DZP 87.2–87.8 NZ 88.6–90.4	50–1600	DZP 21.5 NZ 24.5		[54]
Vitreous humor	LLE + HPLC-DAD	-	DZP 83.8	30–3000	DZP 30.0	DZP 100.0	[55]
Plasma	DLLE + HPLC-DAD	-	-	50–1500	DZP 50.0 NZ 50.0	DZP 60.0 NZ 60.0	[56]
Urine	Oasis MCX-SPE + CE-DAD	-	DZP 78.0	10,000–150,000	DZP 2740	DZP 9140	[57]
Urine	MISPE + HPLC-DAD	OZ	OZ 91.5–92.4 TZ 90.8–91.4 NZ 89.1–89.5 DZP 94.0–99.4	10–1500	OZ 16.2 TZ 21.1 NZ 13.5 DZP 21.0	OZ 53.5 TZ 63.9 NZ 44.5 DZP 69.3	This work

## Data Availability

Data are contained within the article.

## References

[1] Maree R.D., Marcum Z.A., Saghafi E., Weiner D.K., Karp J.F. (2016). A Systematic Review of Opioid and Benzodiazepine Misuse in Older Adults. Am. J. Geriatr. Psychiatry.

[2] Maust D.T., Lin L.A., Blow F.C. (2019). Benzodiazepine Use and Misuse among Adults in the United States. Psychiatr. Serv..

[3] Hood S.D., Norman A., Hince D.A., Melichar J.K., Hulse G.K. (2014). Benzodiazepine dependence and its treatment with low dose flumazenil. Br. J. Clin. Pharmacol..

[4] Longo L.P., Johnson B. (2000). Addiction: Part I. Benzodiazepines—Side effects, abuse risk and alternatives. Am. Fam. Physician.

[5] Diallo S., Bugni E., Senhadj-Raoul F., Gasdeblay S., Marot D., Dessalles M.C., Mahuzier G. (2001). Chromatographic and spectral analytical data for the determination of benzodiazepine abuse in methadone maintenance program. Talanta.

[6] Tanaka E. (2002). Toxicological interactions between alcohol and benzodiazepines. J. Toxicol.-Clin. Toxicol..

[7] Kurzthaler I., Wambacher M., Golser K., Sperner G., Sperner-Unterweger B., Haidekker A., Pavlic M., Kemmler G., Fleischhacker W.W. (2003). Alcohol and/or benzodiazepine use in injured road users. Hum. Psychopharmacol.-Clin. Exp..

[8] Jones A.W., Holmgren A., Holmgren P. (2004). High concentrations of diazepam and nordiazepam in blood of impaired drivers: Association with age, gender and spectrum of other drugs present. Forensic Sci. Int..

[9] Pariente A., Dartigues J.-F., Benichou J., Letenneur L., Moore N., Fourrier-Reglat A. (2008). Benzodiazepines and injurious falls in community dwelling elders. Drugs Aging.

[10] Cheze M., Duffort G., Deveaux M., Pepin G. (2005). Hair analysis by liquid chromatography-tandem mass spectrometry in toxicological investigation of drug-facilitated crimes: Report of 128 cases over the period June 2003 May 2004 in metropolitan Paris. Forensic Sci. Int..

[11] Birkler R.I.D., Telving R., Ingemann-Hansen O., Charles A.V., Johannsen M., Andreasen M.F. (2012). Screening analysis for medicinal drugs and drugs of abuse in whole blood using ultra-performance liquid chromatography time-of-flight mass spectrometry (UPLC-TOF-MS)-Toxicological findings in cases of alleged sexual assault. Forensic Sci. Int..

[12] Bushnell G.A., Gerhard T., Keyes K., Hasin D., Cerda M., Olfson M. (2022). Association of Benzodiazepine Treatment for Sleep Disorders with Drug Overdose Risk among Young People. JAMA Netw. Open.

[13] Bachhuber M.A., Hennessy S., Cunningham C.O., Starrels J.L. (2016). Increasing Benzodiazepine Prescriptions and Overdose Mortality in the United States, 1996–2013. Am. J. Public Health.

[14] Abrahamsson T., Berge J., Ojehagen A., Hakansson A. (2017). Benzodiazepine, z-drug and pregabalin prescriptions and mortality among patients in opioid maintenance treatment—A nation-wide register-based open cohort study. Drug Alcohol Depend..

[15] Mattiuzzi C., Sanchis-Gomar F., Lippi G. (2022). Benzodiazepines consumption may have increased during the COVID-19 pandemic. J. Affect. Disord..

[16] de Dios C., Fernandes B.S., Whalen K., Bandewar S., Suchting R., Weaver M.F., Selvaraj S. (2021). Prescription fill patterns for benzodiazepine and opioid drugs during the COVID-19 pandemic in the United States. Drug Alcohol Depend..

[17] Campitelli M.A., Bronskill S.E., Maclagan L.C., Harris D.A., Cotton C.A., Tadrous M., Gruneir A., Hogan D.B., Maxwell C.J. (2021). Comparison of Medication Prescribing before and after the COVID-19 Pandemic Among Nursing Home Residents in Ontario, Canada. JAMA Netw. Open.

[18] Zaki N., Brakoulias V. (2022). The impact of COVID-19 on benzodiazepine usage in psychiatric inpatient units. Australas. Psychiatry.

[19] Arnhard K., Schmid R., Kobold U., Thiele R. (2012). Rapid detection and quantification of 35 benzodiazepines in urine by GC-TOF-MS. Anal. Bioanal. Chem..

[20] de Bairros A.V., de Almeida R.M., Pantaleao L., Barcellos T., Moura e Silva S., Yonamine M. (2015). Determination of low levels of benzodiazepines and their metabolites in urine by hollow-fiber liquid-phase microextraction (LPME) and gas chromatography-mass spectrometry (GC-MS). J. Chromatogr. B-Anal. Technol. Biomed. Life Sci..

[21] Švidrnoch M., Boráňová B., Tomková J., Ondra P., Maier V. (2018). Simultaneous determination of designer benzodiazepines in human serum using non-aqueous capillary electrophoresis–tandem mass spectrometry with successive multiple ionic–polymer layer coated capillary. Talanta.

[22] Salomone A., Gerace E., Brizio P., Gennaro M.C., Vincenti M. (2011). A fast liquid chromatography-tandem mass spectrometry method for determining benzodiazepines and analogues in urine. Validation and application to real cases of forensic interest. J. Pharm. Biomed. Anal..

[23] Montenarh D., Wernet M.P., Hopf M., Maurer H.H., Schmidt P.H., Ewald A.H. (2014). Quantification of 33 antidepressants by LC-MS/MS-comparative validation in whole blood, plasma, and serum. Anal. Bioanal. Chem..

[24] Wachełko O., Szpot P., Tusiewicz k Nowak K., Chłopaś-Konowałek A., Zawadzki M. (2023). An ultra-sensitive UHPLC-QqQ-MS/MS method for determination of 54 benzodiazepines (pharmaceutical drugs, NPS and metabolites) and z-drugs in biological samples. Talanta.

[25] Fernandez P., Lago M., Alvarez I., Carro A.M., Lorenzo R.A. (2013). Chromatographic determination of benzodiazepines in vitreous humor after microwave-assisted extraction. Anal. Methods.

[26] Rezaei F., Yamini Y., Moradi M., Daraei B. (2013). Supramolecular solvent-based hollow fiber liquid phase microextraction of benzodiazepines. Anal. Chim. Acta.

[27] Shahraki S., Ahmar H., Nejati-Yazdinejad M. (2018). Electrochemical determination of nitrazepam by switchable solvent-based liquid-liquid microextraction combined with differential pulse voltammetry. Microchem. J..

[28] Herrera-Chacon A., Torabi F., Faridbod F., Ghasemi J.B., Gonzalez-Calabuig A., del Valle M. (2019). Voltammetric Electronic Tongue for the Simultaneous Determination of Three Benzodiazepines. Sensors.

[29] Tejedor A.M.G., Fernandez Hernando P., Durand Alegria J.S. (2007). A rapid fluorimetric screening method for the 1,4-benzodiazepines: Determination of their metabolite oxazepam in urine. Anal. Chim. Acta.

[30] Furugen A., Nishimura A., Kobayashi M., Umazume T., Narumi K., Iseki K. (2019). Quantification of eight benzodiazepines in human breastmilk and plasma by liquid-liquid extraction and liquid-chromatography tandem mass spectrometry: Application to evaluation of alprazolam transfer into breastmilk. J. Pharm. Biomed. Anal..

[31] Fernandez P., Vazquez C., Lorenzo R.A., Carro A.M., Bermejo A.M. (2010). Development of a liquid chromatographic method for the simultaneous determination of six benzodiazepines in human plasma after solid-phase extraction. Anal. Lett..

[32] Zhang L., Wu P., Jin Q., Hu Z., Wang J. (2018). Multi-residue analysis of sedative drugs in human plasma by ultra-high performance liquid chromatography tandem mass spectrometry. J. Chromatogr. B-Anal. Technol. Biomed. Life Sci..

[33] Zhao T., Du L., Zhang Z., Li N., Wang M., Ren Q. (2020). A poly(N,N-dimethylaminoethyl methacrylate-co-ethylene glycol dimethacrylate) monolith for direct solid-phase extraction of benzodiazepines from undiluted human urine. Anal. Methods.

[34] Ahmad S.M., Nogueira J.M.F. (2019). High throughput bar adsorptive microextraction: A novel cost-effective tool for monitoring benzodiazepines in large number of biological samples. Talanta.

[35] Alizadeh R., Salami M., Seidi S. (2018). A silica fiber coated with a ZnO-graphene oxide nanocomposite with high specific surface for use in solid phase microextraction of the antiepileptic drugs diazepam and oxazepam. Microchim. Acta.

[36] de Carvalho Abrao L.C., Figueiredo E.C. (2019). A new restricted access molecularly imprinted fiber for direct solid phase microextraction of benzodiazepines from plasma samples. Analyst.

[37] Buzancic I., Pejakovic T.I., Hadziabdic M.O. (2022). A Need for Benzodiazepine Deprescribing in the COVID-19 Pandemic: A Cohort Study. Pharmacy.

[38] Lennestal R., Lakso H.-A., Nilsson M., Mjorndal T. (2008). Urine monitoring of diazepam abuse—New intake or not?. J. Anal. Toxicol..

[39] Umezawa H., Lee X.-P., Arima Y., Hasegawa C., Marumo A., Kumazawa T., Sato K. (2008). Determination of diazepam and its metabolites in human urine by liquid chromatography/tandem mass spectrometry using a hydrophilic polymer column. Rapid Commun. Mass Spectrom..

[40] Andrade-Eiroa A., Canle M., Leroy-Cancellieri V., Cerda V. (2016). Solid-phase extraction of organic compounds: A critical review (Part II). Trac-Trends Anal. Chem..

[41] Hansen F.A., Pedersen-Bjergaard S. (2020). Emerging Extraction Strategies in Analytical Chemistry. Anal. Chem..

[42] Beltran A., Borrull F., Cormack P.A.G., Marce R.M. (2010). Molecularly-imprinted polymers: Useful sorbents for selective extractions. Trac-Trends Anal. Chem..

[43] Kriz D., Ramstrom O., Mosbach K. (1997). Molecular imprinting—New possibilities for sensor technology. Anal. Chem..

[44] Whitcombe M.J., Vulfson E.N. (2001). Imprinted polymers. Adv. Mater..

[45] Ariffin M.M., Miller E.I., Cormack P.A.G., Anderson R.A. (2007). Molecularly imprinted solid-phase extraction of diazepam and its metabolites from hair samples. Anal. Chem..

[46] Hasanah A.N., Soni D., Pratiwi R., Rahayu D., Megantara S., Mutakin (2020). Synthesis of Diazepam-Imprinted Polymers with Two Functional Monomers in Chloroform Using a Bulk Polymerization Method. J. Chem..

[47] Hasanah A.N., Susanti I., Marcellino M., Maranata G.J., Saputri F.A., Pratiwi R. (2021). Microsphere molecularly imprinted solid-phase extraction for diazepam analysis using itaconic acid as a monomer in propanol. Open Chem..

[48] Bakhshi A., Daryasari A.P., Soleimani M. (2021). A Molecularly Imprinted Polymer as the Adsorbent for the Selective Determination of Oxazepam in Urine and Plasma Samples by High-Performance Liquid Chromatography with Diode Array Detection. J. Anal. Chem..

[49] Varenne F., Kadhirvel P., Bosman P., Renault L., Combes A., Pichon V. (2022). Synthesis and characterization of molecularly imprinted polymers for the selective extraction of oxazepam from complex environmental and biological samples. Anal. Bioanal. Chem..

[50] Meatherall R. (1994). Optimal enzymatic-hydrolysis of urinary benzodiazepine conjugates. J. Anal. Toxicol..

[51] Turiel E., Martin-Esteban A. (2010). Molecularly imprinted polymers for sample preparation: A review. Anal. Chim. Acta.

[52] He C., Long Y., Pan J., Li K., Liu F. (2007). Application of molecularly imprinted polymers to solid-phase extraction of analytes from real samples. J. Biochem. Biophys. Methods.

[53] Bui B.T.S., Haupt K. (2010). Molecularly imprinted polymers: Synthetic receptors in bioanalysis. Anal. Bioanal. Chem..

[54] Su Q., Zeng C., Tang Y., Finlow D.E., Cao M. (2012). Evaluation of diazepam-molecularly imprinted microspheres for the separation of diazepam and its main metabolite from body fluid samples. J. Chromatogr. Sci..

[55] Bazmi E., Behnoush B., Akhgari M., Bahmanabadi L. (2016). Quantitative analysis of benzodiazepines in vitreous humor by high-performance liquid chromatography. SAGE Open Med..

[56] Saldanhaa G.A., Bezerra A.L., Fonseca A.M., Valle A. (2022). Analysis of benzodiazepines in plasma samples by dllme and lc-dad: Critical aspects, flaws and issues encountered—A discussion. Quim. Nova..

[57] Su H.L., Kao W.C., Lin K.W., Lee C.Y., Hsieh Y.Z. (2010). 1-Butyl-3-methylimidazolium-based ionic liquids and an anionic surfactant: Excellent background electrolyte modifiers for the analysis of benzodiazepines through capillary electrophoresis. J. Chromatogr. A.

[58] Mandrioli R., Mercolini L., Raggi M.A. (2008). Benzodiazepine Metabolism: An Analytical Perspective. Curr. Drug Metab..

[59] Chouinard G., Lefko-Singh K., Teboul E. (1999). Metabolism of anxiolytics and hypnotics: Benzodiazepines, buspirone, zoplicone, and zolpidem. Cell. Mol. Neurobiol..

